# Color morphology of *Diaphorina citri* influences interactions with its bacterial endosymbionts and ‘*Candidatus* Liberibacter asiaticus’

**DOI:** 10.1371/journal.pone.0216599

**Published:** 2019-05-16

**Authors:** Saeed Hosseinzadeh, John Ramsey, Marina Mann, Lily Bennett, Wayne B. Hunter, Masoud Shams-Bakhsh, David G. Hall, Michelle Heck

**Affiliations:** 1 Boyce Thompson Institute, Ithaca, NY, United States of America; 2 Department of Plant Pathology, Tarbiat Modares University, Tehran, Iran; 3 USDA ARS Emerging Pests and Pathogens Research Unit, Robert W. Holley Center for Agriculture and Health, Ithaca, NY, United States of America; 4 US Department of Agriculture, Agricultural Research Service, Fort Pierce, FL, United States of America; 5 Plant Pathology and Plant Microbe Biology Section, School of Integrative Plant Science, Cornell University, Ithaca, NY, United States of America; Southeastern Louisiana University, UNITED STATES

## Abstract

*Diaphorina citri* is a vector of ‘*Candidatus* Liberibacter asiaticus,*’* (*C*Las), associated with Huanglongbing, (HLB, or citrus greening) disease in citrus. *D*. *citri* exhibits three different color morph variants, blue, gray and yellow. Blue morphs have a greater capacity for long-distance flight as compared to non-blue morphs, but little else is known about how color morphology influences vector characteristics. In this study, we show that the color morphology of the insect is derived from pigmented cells of the fat body. Blue morphs acquire a lower level of *C*Las in their bodies from infected trees as compared to their gray and yellow conspecifics, referred to in this paper collectively as non-blue morphs. Accordingly, *C*Las titer in citrus leaves inoculated by non-blue insects was 6-fold higher than in leaves inoculated by blue insects. Blue color morphs harbored lower titers of *Wolbachia* and ‘*Candidatus* Profftella armatura,’ two of the *D*. *citri* bacterial endosymbionts. Expression of hemocyanin, a copper-binding oxygen transport protein responsible for the blue coloration of hemolymph of other arthropods and mollusks, was previously correlated with blue color morphology and is highly up-regulated in insects continuously reared on *C*Las infected citrus trees. Based on our results, we hypothesized that a reduction of hemocyanin expression would reduce the *D*. *citri* immune response and an increase in the titer of *C*Las would be observed. Surprisingly, a specific 3-fold reduction of hemocyanin-1 transcript levels using RNA silencing in blue adult *D*. *citri* morphs had an approximately 2-fold reduction on the titer of *C*Las. These results suggest that hemocyanin signaling from the fat body may have multiple functions in the regulation of bacterial titers in *D*. *citri*, and that hemocyanin is one of multiple psyllid genes involved in regulating *C*Las titer.

## Introduction

The Asian citrus psyllid, *Diaphorina citri* Kuwayama, is an economically important pest of citrus primarily because it is a vector of ‘*Candidatus* Liberibacter asiaticus’ (*C*Las). *C*Las is a phloem-limited, gram-negative, fastidious bacterium that is associated with the most serious disease of citrus, huanglongbing (HLB), also known as citrus greening disease. *D*. *citri* and HLB have spread to most citrus growing regions worldwide. In the USA, HLB was first detected in 2005 in Florida. Today, every citrus growing county in the state of Florida harbors infected trees. The disease jeopardizes the future of the state’s annual, nine-billion-dollar industry. *C*Las and *D*. *citri* have been detected in California and in Texas’ Rio Grande Valley. Detection of HLB in California rose a startling 160% in 2018 [1]. The need for novel and effective HLB management strategies is urgent.

There is no cure for HLB, which renders citrus trees unproductive and ultimately kills the trees. HLB disease management options are currently limited to strategies that reduce inoculum loads in a grove (vector control, identifying and removing infected trees, and replanting with disease-free trees) and accurate and sensitive early detection methods to expedite removal of infected trees [2]. Current management strategies are ineffective for several reasons. Urban cultivation of citrus and other hosts of *D*. *citri* near commercial groves in affected areas is a primary cause of concern. Cultural control measures such as crop-rotation, using pest resistant varieties and intercropping with pest–repellent plants are not feasible options for controlling *D*. *citri* [3]. Long-term management of HLB using insecticides is not effective at stopping the spread of HLB because insect transmission of the pathogen occurs even under insecticide pressure. Blocking the spread of *C*Las by disrupting transmission of the bacteria by the insect vector could be a new approach in the fight against HLB and other insect vector-borne plant diseases [4].

Like other hemipteran insects, *D*. *citri* has established cooperative associations with obligate bacterial endosymbionts including ‘*Candidatus* Carsonella ruddii’, ‘*Candidatus* Profftella armatura’[5] and *Wolbachia pipientis* wDi [6] during its life history. Obligate symbionts have been shown to be important for insect reproduction, behavior and pigmentation [7–9]. A large proportion, 15%, of the reduced genome of *Profftella* is devoted to polyketide synthase (PKS) biosynthetic gene clusters which produce a polyketide, diaphorin, with unknown function in the biology of *D*. *citri* [5]. Notably, 17.6% of open reading frames of *Carsonella* are devoted to amino acid metabolism, and it is expected that this endosymbiont performs a similar function as *Buchnera aphidicola*, the symbiont of aphids, namely to supply essential amino acids lacking in the phloem sap diet for its insect host [10]. Additionally, *Wolbachia* has been considered unique in its ability to alter reproduction of its hosts through induction of feminization, parthenogenesis, male killing, and cytoplasmic incompatibility [11], but little is known about the function of *Wolbachia* in *D*. *citri*. Recently, a small DNA-binding protein encoded by *Wolbachia* was identified as the repressor of the *C*Las phage holin promoter, indicating potential involvement of this bacterium in suppression of the lytic cycle of SC1 prophage in *D*. *citri* [12].

*C*Las transmission by *D*. *citri* has been defined as circulative and propagative [13], and is comprised of four main steps: acquisition, replication, translocation and transmission. *C*Las must be acquired from an infected tree during feeding, after which it must cross the gut barrier. Once acquired through the gut tissue, *C*Las moves through the hemolymph until passing the salivary gland membranes, where it is inoculated into a new host plant together with the insect’s salivary secretions. During this circulative journey, *C*Las must evade the insect’s immune response [13–19]. Gut and hemolymph proteomic analysis shows distinct interactions between *C*Las and these tissues in the insect during circulative transmission [14, 19]. Transmission refers to the process of inoculation of a new host tree by a psyllid that has acquired the bacterium. Transmission of *C*Las by adults is more efficient when bacteria are acquired by nymphs [13, 18].

Whole insect proteomic studies have enabled the identification of proteins differentially expressed when adult and nymph *D*. *citri* are reared on *C*Las -infected trees [20, 21]. However, since *C*Las is unculturable, it is impossible to disentangle the indirect effects of the *C*Las-infected tree on the *D*. *citri* proteome from direct effects of *C*Las in the vector’s body in those studies. Consequently, although we have an in-depth understanding of how *D*. *citri* responds to *C*Las infection in the tree, the *D*. *citri* genes and proteins performing a direct function in regulating *C*Las acquisition and transmission remain elusive. The difficulty in pinpointing which proteins are involved in *C*Las transmission and how they may be functioning (to either promote or inhibit transmission) is confounded by the fact that a relatively low percentage of adult *D*. *citri* actually transmit the pathogen although a much greater proportion of *D*. *citri* can be infected with *C*Las as evidenced by qPCR assays [13]. Additionally, not all populations of *D*. *citri* acquire and transmit the bacterium with the same efficiency [22]. Acquisition and transmission efficiencies are heritable traits in *D*. *citri*, proving a genetic basis for the regulation of these phenotypes [22].

From the proteome studies [20], one of the most highly expressed proteins in *C*Las-exposed nymph and adult *D*. *citri* is a hemocyanin, an oxygen transport protein with functions in immunity and defense reported in other arthropods [23]. The C-terminus of *D*. *citri* hemocyanin-1 was shown to physically interact with the *C*Las coenzyme A (CoA) biosynthesis enzyme phosphopantothenoylcysteine synthetase/decarboxylase [20]. Hemocyanins are respiratory proteins with conserved histidine residues forming a coordination complex with copper ions to bind oxygen for transport in the hemolymph [24]. The expression of hemocyanin is correlated to color morphology in *D*. *citri* [20]. There are at least three *D*. *citri* color morphs: blue, gray and yellow. Our lab has previously shown that hemocyanin transcript expression in blue morphs is >3 fold greater than in gray and yellow *D*. *citri* [20]. Based on expression differences of hemocyanin in these color morphs, we grouped them as blue or non-blue color (comprised of gray or yellow) morphs for the follow-up research presented in this paper. Blue *D*. *citri* have enhanced flight capabilities, and coupled to our hemocyanin expression data, we hypothesize that these data suggest the greater levels of hemocyanin in the blue color morphs provide the insect with an enhanced metabolic capacity that could benefit vector performance and transmission. Hemocyanin may also play a role in *D*. *citri* immunity against *C*Las. The role of hemocyanin in arthropod innate immunity has been previously documented, including the conversion of hemocyanin into phenoloxidase [25]. An antimicrobial peptide derived from the C-terminus of crayfish, hemocyanin was shown to inhibit the growth of both gram-negative and gram-positive bacteria [23].

Because not all insects within a population of *D*. *citri* acquire or transmit *C*Las, and because the proteomic analysis was conducted on pools of *C*Las-exposed insects collected from HLB positive trees, the role of hemocyanin in acquisition and transmission is unknown. The excellent correlation of hemocyanin expression level to color morphology in our psyllid population and the use of RNA silencing tools paved the way for studies examining differences in *C*Las-vector relations among the different *D*. *citri* color morphs and hemocyanin expression levels. Results from these studies show an interplay between color morphology, hemocyanin and molecular interactions among *D*. *citri*, its endosymbionts and *C*Las.

## Materials and methods

### Insect rearing

*D*. *citri* colonies were maintained on both *C*Las-infected and uninfected sweet orange (*Citrus sinensis* “Madam Vinous”) and citron (*Citrus medica*). Plants and insects were maintained in growth chambers set at 26°C and 70% relative humidity with photoperiods of 14:10 h (light:dark). Adults were age-synchronized to 5–7 days and collected using a vacuum pump in 50 ml Corning falcon tubes. Harvested insects were kept on ice and inspected under a stereomicroscope to determine their abdominal color within one week of molting to the adult stage. For gene expression analysis, quantitative PCR (qPCR), and western blotting, insects were sorted into four groups according to their abdominal color: blue, yellow, gray, and intermediate (with the intermediate group comprising the approximately 10–20% of adult insects which could not be clearly grouped by color). For acquisition and transmission assays, yellow and gray insects were grouped together as non-blue insects. Insects reared on healthy citrus trees (which also tested negative for *C*Las by qPCR) are called non-exposed insects. Insects which are reared on infected trees and test positive by qPCR are referred to as *C*Las–exposed.

### Imaging of blue and non-blue adults

Five each, mixed-sex, blue and non-blue adult *D*. *citri* raised on non-infected citron were chilled on ice before being dissected in 1xPBS under an epifluorescence microscope (Leica DM5500, Wetzlar, Hesse, Germany). Dissections were performed live, non-fixed, and chilled. Images were taken using a color CCD camera Retiga-2000R connected to the Qcapture Pro 6.0 acquisition software.

### Size and body mass among different color morphs

Age-synchronized insects reared on *C*Las-infected sweet orange plants were collected by a vacuum pump in 50 ml falcon tubes and were kept on ice to differentiate their sex and color morph using a stereomicroscope. Twenty male and female adults of each color morph (blue, yellow, gray) were weighed individually using an analytical microbalance with a sensitivity of 0.0001 mg (Sartorius SE2, Sartorius AG, Göttingen, Germany).

### *D*. *citri* endosymbiont copy number quantification by qPCR

Non-exposed *D*. *citri* were collected from sweet orange plants, and a stereomicroscope (AmScope, SM-1BZ-FRL) was used to classify the abdominal color of each insect as blue or non-blue within one week of molting to the adult stage. *D*. *citri* samples were frozen and subjected to cryogenic lysis. Total DNA was isolated using the DNeasy blood and tissue kit (Qiagen, Hilden, Germany). DNA concentration was determined using a Nanodrop (Thermo Fisher Scientific, Waltham, MA, USA), and 100 ng of DNA was used per PCR reaction. The Applied Biosystems QuantStudio 6 Flex Real-Time instrument was used for qPCR analysis. Endosymbiont qPCR was performed using the Fast SYBR Green Master Mix (Life Technologies, Carlsbad, CA, USA) and primer sequences from [26]. All qPCR reactions were performed in triplicate and following thermal cycling programs: [20 seconds at 95°C; 40 cycles of (3 seconds at 95°C; 30 seconds at 60°C)]. Approximation of microbial copy number was enabled by comparing Ct values from biological samples to Ct values from standard curves made using serial dilutions of synthetic plasmids containing qPCR target region.

### Acquisition and detached leaf transmission assays

All leaves used in the experiment were harvested from one-year-old sweet orange or citron seedlings grown in a greenhouse maintained at 21°C with a photoperiod of 12 hours. The experiments were conducted as described by Ammar *et al*. [27] with the following modifications. Fully expanded young dark green leaves (5–6 cm x 2.5–3.5 cm) were cut by razor blade so that about 2 cm of the stem remained attached to the leaves [28]. Leaves attached to a stem fragment were placed in a 2 ml sterile tube containing 2 ml sterile water and were contained within 50 ml centrifuge tubes. Ten blue or non-blue sweet orange or citron-reared adult *D*. *citri* less than one week after molting were placed on each leaf, and all tubes were maintained in the same growth chambers used for insect colony maintenance. After two weeks, experimental insects were removed from leaves and were flash frozen. These insects were processed for the analysis of *C*Las acquisition by qPCR for *C*Las detection. Leaf midribs were dissected and frozen in liquid nitrogen for *C*Las titer determination by qPCR for transmission assays. Each experiment was repeated twice each time with 15 biological replicate transmission assays for each color morph.

### Citrus leaf midrib and insect DNA extraction and qPCR

Flash frozen citrus leaf midribs were ground to a fine powder in a chilled mortar and pestle and transferred into a 2 ml tube. Total DNA extraction from plant tissue was performed using the DNeasy Plant Mini Kit (Qiagen, Hilden, Germany). For evaluation of *C*Las titer in insects, flash frozen insects were cryoground using a mixer mill (Retsch Mixer Mill 400, Haan, Germany), and total DNA was extracted by the DNeasy blood and tissue kit (Qiagen, Hilden, Germany) and quantified by Nanodrop. *C*Las qPCR was performed using the Taqman Universal qPCR Master Mix (Life Technologies, Carlsbad, CA, USA) and primer and probe sequences from Ramsey et al. [21]. The Applied Biosystems QuantStudio 6 Flex Real-Time instrument was used for qPCR analysis, with samples analyzed in triplicate using the following thermal cycling programs: [10 minutes at 95°C; 40 cycles of (15 seconds at 95°C, 60 seconds at 60°C)]. For leaf midrib samples, 50 ng of DNA were used for each reaction; for insect samples, 200 ng of DNA were used for each reaction. Each biological sample was run in technical triplicate. Standard curves were generated from the mean Ct values of serial dilutions of a synthetic plasmid containing the qPCR target region and this standard curve was used to estimate the *C*Las copy number by putting the observed Ct value for the samples into the formula: (observed Ct − y intercept)/slope.

### *D*. *citri* hemocyanin antibody production

We used western blot analysis to measure hemocyanin protein in *D*. *citri*. An affinity purified anti-hemocyanin polyclonal antibody was produced by Genscript in rabbit using a peptide immunogen from the hemocyanin C domain of the *D*. *citri* hemocyanin-1 protein (Genebank ID XP_008477906.1). The XP_008477906.1 sequence has now been replaced with XP_017301848.1 by Genbank as part of standard genome annotation processing. The peptide antigen spanned amino acids 855–868 of the protein, with a cysteine residue added at the N-terminus to facilitate conjugation of the peptide to the carrier protein (CR^855^NSHEFTEASDEAP^868^). Specificity of the antibody was validated by western blot analysis.

### SDS-PAGE and western blot analysis

Fifty adult insects per sample were used for protein extractions for western blot analysis of hemocyanin expression in blue and non-blue *D*. *citri*. Insects were flash frozen and cryoground (Retsch Mixer Mill 400, Haan, Germany) in 2 ml microcentrifuge tubes containing three small 3.2 mm stainless steel beads (Next Advance, Averill Park, NY, USA). The insect powder was suspended in 100 μl lysis buffer [70 mM Tris (pH 6.8), 12% glycerol, 1.2x HALT protease inhibitor)], and the samples were mixed and centrifuged briefly. The lysate was transferred into a new 1.5 ml microcentrifuge tube and disrupted for 30 seconds at 15% amplitude using a probe sonicator (Branson 450 Digital Sonifier, Danbury, CT, USA) on ice to yield a yellow-brown lysate. The resulting insect lysate was centrifuged at 13,000 rpm for 15 sec and the clear yellow supernatant was transferred into new tubes. Protein concentration was estimated in samples by Quick Start Bradford protein assay (Bio-Rad, Hercules, CA, USA). The amount of lysate containing 20 μg of protein was transferred into a new tube and suspended with running buffer to a final concentration of 2% SDS, 0.02% bromophenol blue, and 2.5% β-mercaptoethanol. Sample volume was adjusted to 40 μl with 1x PBS which contained 20 μg of protein. Samples were boiled for 5 min at 95°C and analyzed on Mini-Protean TGX pre-cast protein gels (10%) (Bio-Rad, Hercules, CA, USA). Separated proteins were transferred to a 0.45 μm Nitrobind Pure Nitrocellulose membrane (GE Water and Process Technologies, Trevose, PA, USA). Nonspecific antibody binding sites on the membranes were blocked by overnight incubation with gentle agitation in 5% instant nonfat dry milk (Best Yet, Bethpage, NY) in 1X Tris-buffered saline with 0.5% Tween-20 (TBST). Membranes were first incubated with the anti-hemocyanin polyclonal antibody (1:1,000 dilution in TBST with 0.1% bovine serum albumin) for one hour, followed by three washes with TBST for 10 minutes each. Membranes were then incubated with the horseradish peroxidase (HRP)-conjugated anti-rabbit IgG secondary antibody (Promega, 1:5000 dilution in TBST with 0.1% bovine serum albumin). Following three washes with TBST (10 minutes each), and one wash with TBS (five minutes), the antigen–antibody complexes were visualized by chemiluminescence using the ECL Detection Reagent (Amersham Life Science, Piscataway, NJ, USA). Western blot analysis of hemocyanin expression in healthy compared to *C*Las-exposed *D*. *citri* was performed using frozen protein samples prepared for mass spectrometry analysis by trichloroacetic acid/acetone precipitation as described [21, 29].

### Elemental copper analysis

Non-exposed adult *D*. *citri* were collected from healthy sweet orange plants, sorted according to their abdominal color on ice using a stereomicroscope, and flash frozen. Insect abdominal color was classified as either blue, gray, or yellow as previously described above. For each of the three color morphs, five samples of 50 pooled adult *D*. *citri* were collected and dried for elemental copper analysis. Insects were placed in glass test tubes and dried in a laboratory oven at 60°C for seven days, and the dry weights of the insects were recorded. The samples were treated with 3.0 ml of 60:40 HNO_3_ and HClO_4_ mixture in a Pyrex glass tube and left overnight at room temperature to destroy organic matter. The mixture was then heated to 120°C for two hours. The temperature of the heating block was then raised to 145°C for two hours. An additional 2 ml of nitric acid was added to eliminate the brownish color of organic matter. The temperature of the heating block was then raised to 190°C for 10 minutes and turned off. The cooled samples in the tubes were then vortexed and transferred into auto sampler tubes to run in Inductively Coupled Plasma Atomic Emission Spectrometer (ICP-AES; Thermo iCAP 6500 series) as described in [30].

### Double stranded (ds) RNA design and synthesis

Three different dsRNA constructs were designed and synthesized (AgroRNA, Seul Korea) to target *D*. *citri* hemocyanin-1 (XM_017446359.1; annotated in Genbank as “allergen Cr-PI-like”) (S1 Table). Based on preliminary experiments to test the efficacy of these three constructs for silencing the hemocyanin-1 gene (not shown), only DC-Hm2R was used in the subsequent experiments. *Chinese sacbrood virus* (CSBV) dsRNA (240 bp) was used as a negative control based on its use in previous RNA silencing experiments in *D*. *citri* [31]. DsRNA was diluted in RT-PCR Grade Water (Ambion, Austin, TX, USA), purified using the MEGAclear kit (Ambion, Austin, TX, USA), quantified by Nanodrop, and diluted in water to a concentration of 1 μg/μl for use in RNA silencing experiments.

### dsRNA microinjection

Adult insects were collected from *C*Las-infected sweet orange plants and blue color morphs were selected by stereomicroscope for microinjection (AmScope, SM-1BZ-FRL, Irvine, CA, USA). The insects were placed in a weighing dish chilled on ice packs for immobilization prior to microinjection. Injection glass pipettes were pulled from Kwik-Fill borosilicate glass capillaries, 1.0 mm × 6″ (World Precision Instruments, Sarasota, FL) with a pipette puller (Sutter Instruments, P-87, Novado, CA, USA).

Approximately 100 ng of dsRNA (100 ng/μl in water) was injected at the side of the thorax, leading to visible swelling of the insect body. The insects were transferred onto healthy sweet orange leaves immediately after microinjection (10 replicate leaves with 10 insects per leaf). Ten days after injection, the insects were collected for analysis of RNA (to evaluate hemocyanin expression level) and DNA (to evaluate *C*Las titer). Insects were cryoground, and the resulting powder was divided into two parts for DNA and RNA extraction. Viability of insects after injection was assessed after 24 hours and was greater than 95% in all experiments.

### Hemocyanin expression in microinjected insects

Total RNA was isolated from cryoground insects using the RNeasy kit (Qiagen, Hilden, Germany). DNA was removed by treatment of samples with RNase-free DNase I (Thermo Fisher Scientific, Waltman, MA, USA), and RNA was quantified by NanoDrop. RNA (1μg) was used for cDNA synthesis by iScript cDNA Synthesis Kit (Bio-Rad, Hercules, CA, USA). The cDNA was diluted three-fold in water and 1 μl of the diluted cDNA was used as template for qPCR. Using qPCR the relative gene expression quantification was performed on the Applied Biosystem QuantStudio 6 using the Fast SYBR Green Master Mix (Life Technologies, Carlsbad, CA, USA). ACP alpha-tubulin was used as a housekeeping control gene for Ct value normalization. Primer sequences used in this study are: hemocyanin (Forward: 5′ CTCCCCAAGGGATCCAGAGA 3′; Reverse: 5′ AAGGACGGTCGAATGGGAAC 3′); tubulin (Forward: 5′ GCGTCTCTTCGGTTTGACGG 3′; Reverse: 5′ CACTTCACCATCTGGTTGGC 3′). The obtained Ct values were converted into normalized relative quantities (NRQs) [32] and the resulting NRQ values were used for statistical analysis.

### Statistical analysis

Data were checked for normality and then the Student’s *t*-test was used for statistical analysis of the following: insect mass; copper concentration and *C*Las and endosymbiont titer in blue or non-blue *D*. *citri*; *C*Las titer in *D*. *citri* after hemocyanin silencing via microinjection of control dsRNA or hemocyanin dsRNA; *C*Las titer in citrus leaves inoculated with blue or non-blue *D*. *citri*. For all analyses, differences among conditions were considered significant when *P* <0.05.

## Results

### *D*. *citri* abdominal color morphology is derived from pigmented cells of the fat body

**Fig 1:**
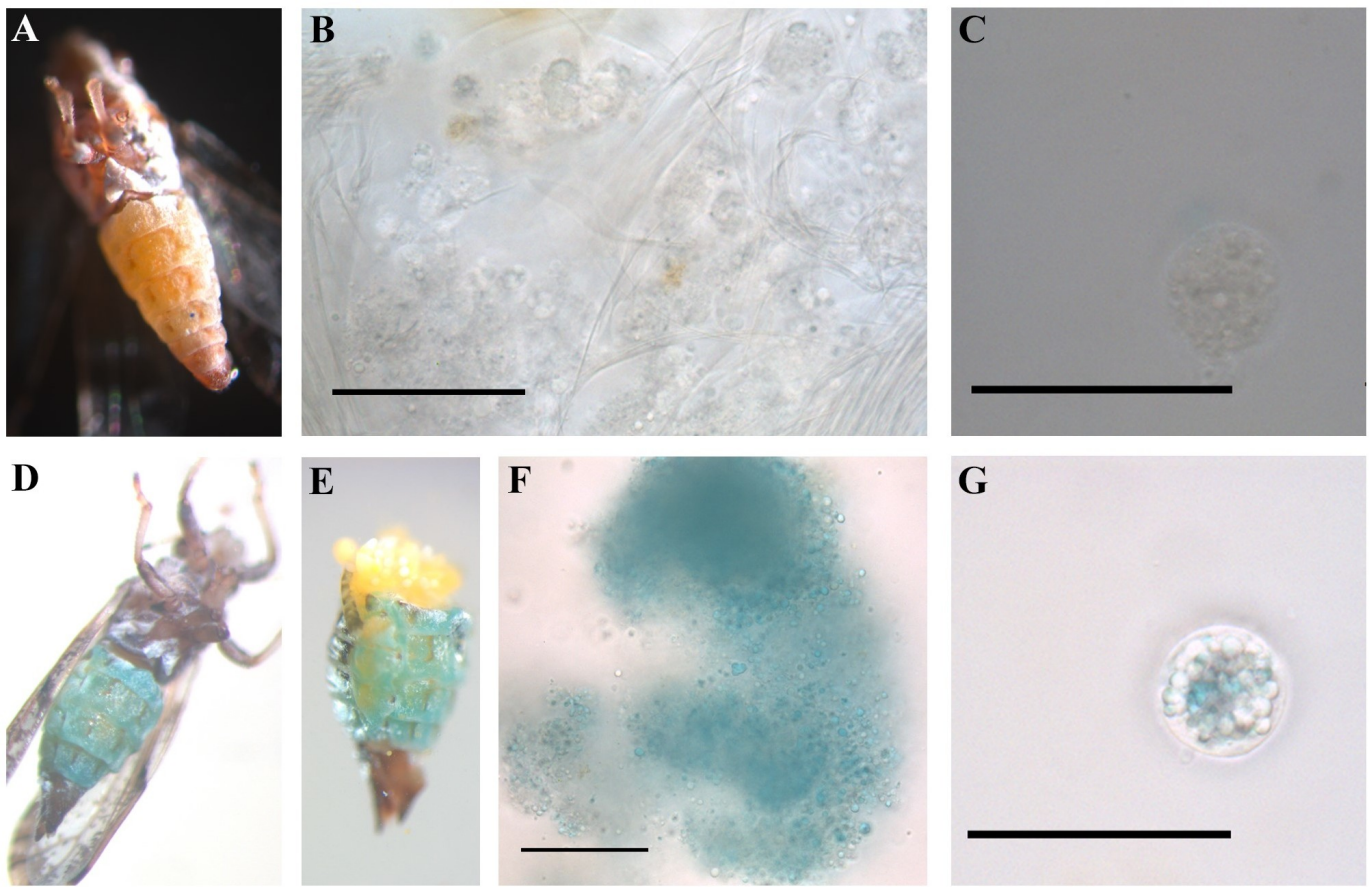
Blue *D*. *citri* body color originates from sub-cellular blue pigmentation of the fat body cells as shown during live dissection imaging. Dissections occurred under natural light in 1xPBS and are not fixed. (A) Ventral view of a non-blue male *D*. *citri*. (B) Fat body cells from a non-blue adult. (C) Single fat body cell from non-blue adult showing droplet-like composition of cell and transparency. (D) Ventral view of blue, adult, female *D*. *citri*. (E) Ventral view of the blue, adult female from (D), after separating the abdomen from the thorax and beginning to remove internal organs. (F) Loose cloud of fat body cells from the blue, adult female in (D), showing higher intensity of blue color in areas with higher concentration of cells, as well as delicate nature of the fat body organ and ephemeral nature of individual cell cohesion. (G) Single fat body cell from blue, adult female in (D), showing droplet-like composition of cells where some droplets are pigmented bright blue, and cells vary in size.

*D*. *citri* has at least three different color morphs, blue, gray and yellow, and each color is observed in the abdomen of the insect. Additionally, the color varies in intensity. Dissection of abdomens from blue adult *D*. *citri* reared on healthy sweet orange reveals that the fat body contains cells varying in size from 20–40 μm in diameter that contain blue pigments–pigments that are absent from the fat body of non-blue adult insects (**Fig 1**). The cells are loosely associated with each other in the fat body and fall apart easily into droplets upon dissection.

### *D*. *citri* color morph influences female but not male body mass

**Fig 2:**
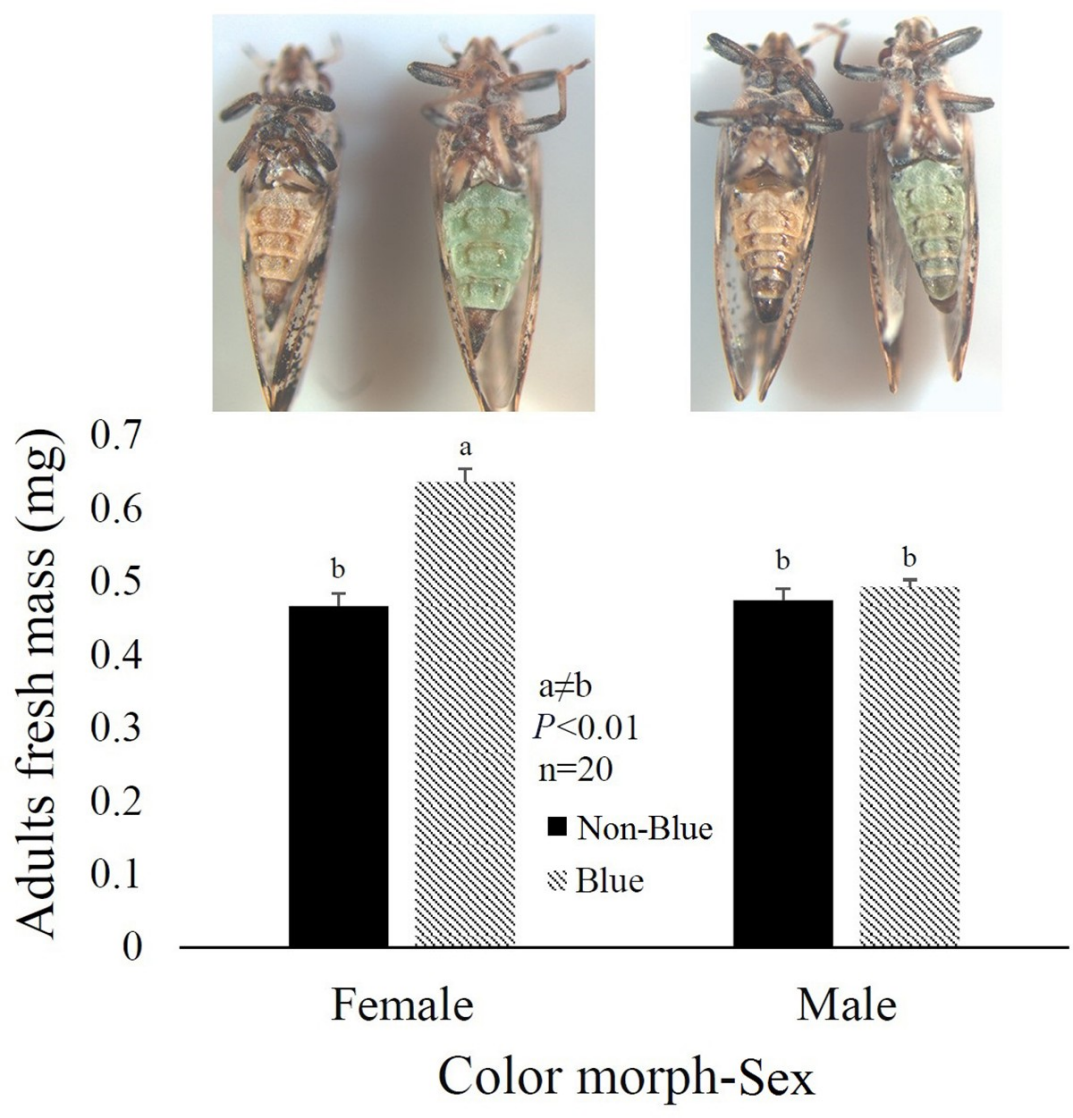
*D*. *citri* body mass by sex and color morph. Blue color morph females have 40% more body mass compared to non-blue color females. Color morph had no effect on male body mass.

Color morph had a pronounced effect on the body mass of female *D*. *citri* (n = 20, *P* <0.01). Adult insects with blue abdominal color reared on healthy sweet orange had 40% more body mass as compared to non-blue females. No significant difference occurred between the body mass of males across different color morphs (**Fig 2**).

### Blue *D*. *citri* color morphs have lower titers of *Wolbachia* and *Profftella* as compared to non-blue morphs

**Fig 3:**
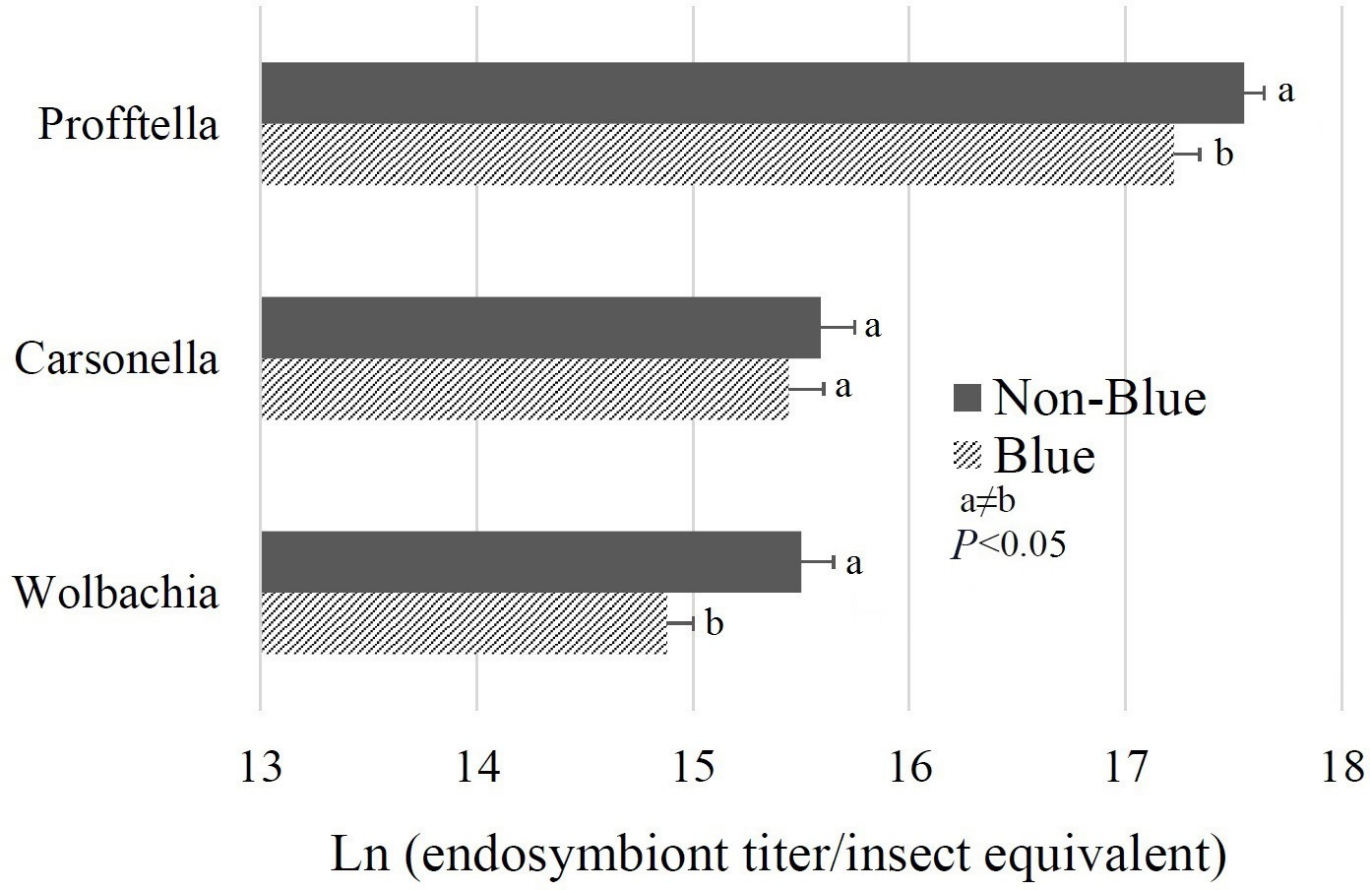
The titer of the bacterial endosymbionts *Profftella*, *Carsonella*, and *Wolbachia* in *C*Las-non-exposed, blue and non-blue adult *D*. *citri*, represented as natural logarithm (endosymbiont titer/insect equivalent). Titer of *Profftella* and *Wolbachia* were higher in non-blue *D*. *citri* as compared to blue *D*. *citri (P* <0.05, n = 10 biological replicates).

The titers of the *D*. *citri* endosymbionts *Profftella*, *Carsonella*, and *Wolbachia* were quantified by qPCR and compared between non-exposed blue and non-blue *D*. *citri* adults. Significantly greater titers of *Wolbachia* and *Profftella* were detected in non-blue color morphs as compared to blue color morphs *(P* = 0.01 and 0.04, respectively, **Fig 3**). No significant difference occurred in *Carsonella* titer between color morphs of adults *(P* = 0.51, **Fig 3**).

### *C*Las acquisition and transmission is significantly higher for non-blue compared to blue adult *D*. *citri* on sweet orange

**Fig 4:**
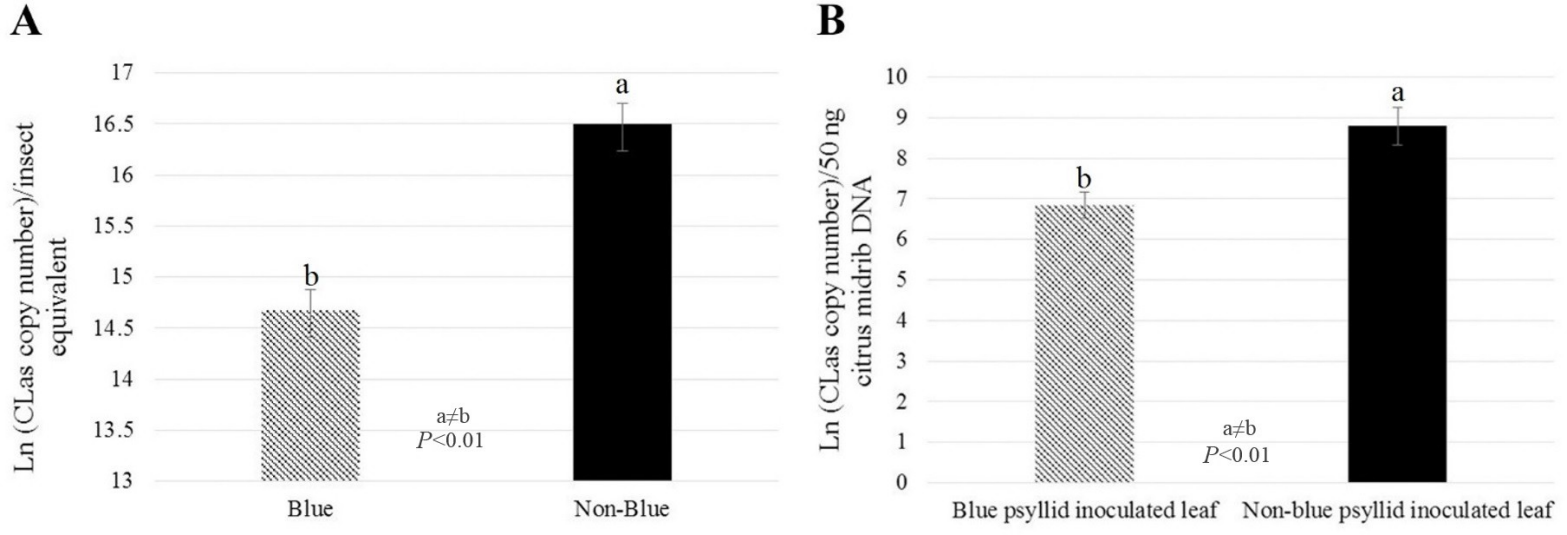
*C*Las titer in blue and non-blue color morphs of *D*. *citri* adults and in citrus leaves inoculated by blue and non-blue insects. (A) *C*Las copy number per insect equivalent is ~8-fold greater in non-blue compared to blue *D*. *citri (P* <0.01). (B) *C*Las copy number in midrib DNA samples from citrus leaves inoculated with non-blue *D*. *citri* is ~6 fold greater than in leaves inoculated with blue *D*. *citri (P* <0.01).

We tested whether color morphology had an impact on *C*Las acquisition and transmission. For these experiments, qPCR was used to estimate *C*Las copy number in DNA samples isolated from insects and dissected midribs inoculated by *C*Las-exposed *D*. *citri*. The *C*Las copy number per insect equivalent was 8-fold greater in non-blue compared to blue sweet orange-reared *D*. *citri (P* <0.01, **Fig 4**). Correspondingly, *C*Las copy number in midrib DNA samples from leaves inoculated with non-blue *D*. *citri* was 6-fold greater than in leaves inoculated with blue *D*. *citri (P* = 0.01, **Fig 4**). In addition, the average *C*Las copy number per insect equivalent in citron-reared color morph was 3.6-fold greater in non-blue compared to blue *D*. *citri*, although due to high variability within sample classes, this difference was not statistically significant (**S1 Fig**). Because this difference was not significant, detached leaf transmission assays were not performed for citron-reared insects.

### Hemocyanin protein and elemental copper are more abundant in blue *D*. *citri* color morphs as compared to non-blue morphs

**Fig 5:**
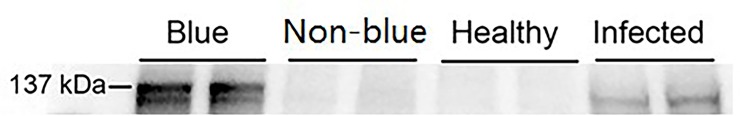
Western blot analysis of hemocyanin protein expression in *D*. *citri*. Twenty μg of protein extracted from each of the four *D*. *citri* samples was used in western blot analysis. Blue: blue color morph adults reared on healthy sweet orange. Non-blue: non-blue color morph adults reared on healthy sweet orange. Healthy: non-exposed adults reared on healthy citron. Infected: *C*Las-exposed adults reared on infected citron.

We used the anti-hemocyanin antibody to investigate the correlation between hemocyanin-1 protein abundance in *D*. *citri* and insect abdominal color, as was previously observed at the mRNA level [20]. We analyzed 20 μg of *D*. *citri* protein samples from blue and non-blue healthy insects by western blot using an affinity purified anti-hemocyanin antibody and an anti-rabbit HRP conjugated secondary antibody. Protein samples from mixed color healthy and *C*Las-exposed *D*. *citri* were also analyzed by western blot. The predicted hemocyanin-1 protein sequence is 1114 amino acids (131 kDa). The first 18 amino acids of the protein were predicted by SignalP [33] to constitute a signal peptide, and the molecular weight of the 996-amino acid processed protein is 129 kDa. Chemiluminescent detection revealed that the most prominent band on the western blot measures between 120–130 kDa, which corresponds to the molecular weight of *D*. *citri* hemocyanin-1. Signal intensity was dramatically greater in the gel lane with protein extracts from *C*Las-exposed *D*. *citri* compared to non-exposed *D*. *citri*. In addition, the chemiluminescent signal was much more intense from the blue compared to non-blue *D*. *citri* protein extract, with the difference even more intense than the difference between non-exposed and *C*Las-exposed *D*. *citri* (**Fig 5**).

**Fig 6:**
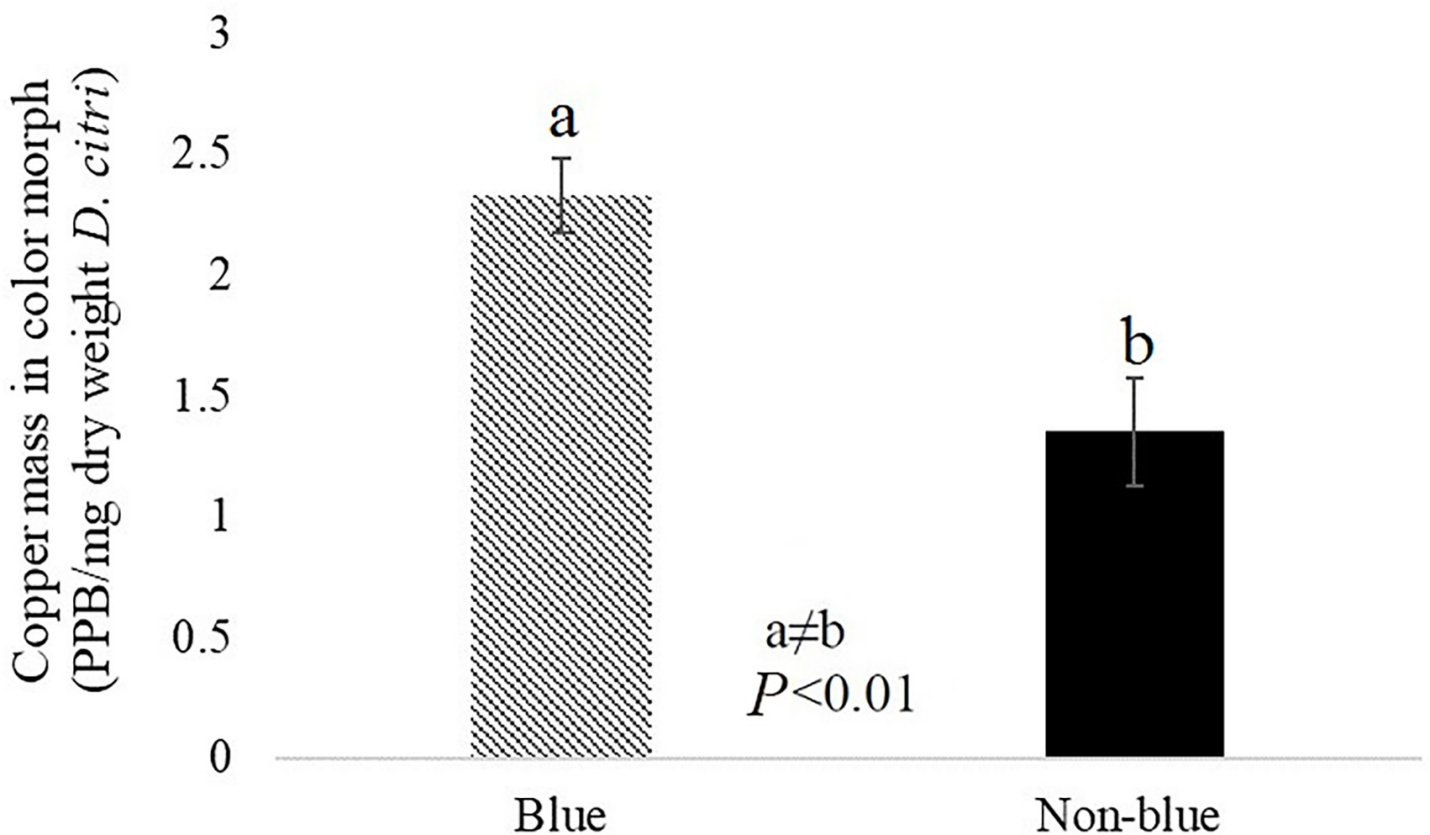
Comparison of copper mass between color morph *D*. *citri* adults using ICP mass spectrometry.

Hemocyanin is a metal binding protein containing copper atoms that reversibly bind oxygen. Elemental copper analysis on blue and non-blue adult *D*. *citri* was performed using an Inductively Coupled Plasma Atomic Emission Spectrometer (ICP-AES, Thermo Electron corporation, US). Significantly more copper was found in blue compared to non-blue *D*. *citri*, both on a per insect basis (blue: 22.67 copper PPB/50; non-blue: 12.68 copper PPB/50 *D*. *citri*) and on a per insect dry weight basis (blue: 2.32 copper PPB/mg; non-blue: 1.42 copper PPB/mg *D*. *citri*, *P* <0.01, n = 5, **Fig 6**).

### Microinjection of hemocyanin-1 dsRNA into adult *D*. *citri* downregulated the expression of hemocyanin-1 by 3-fold

**Fig 7:**
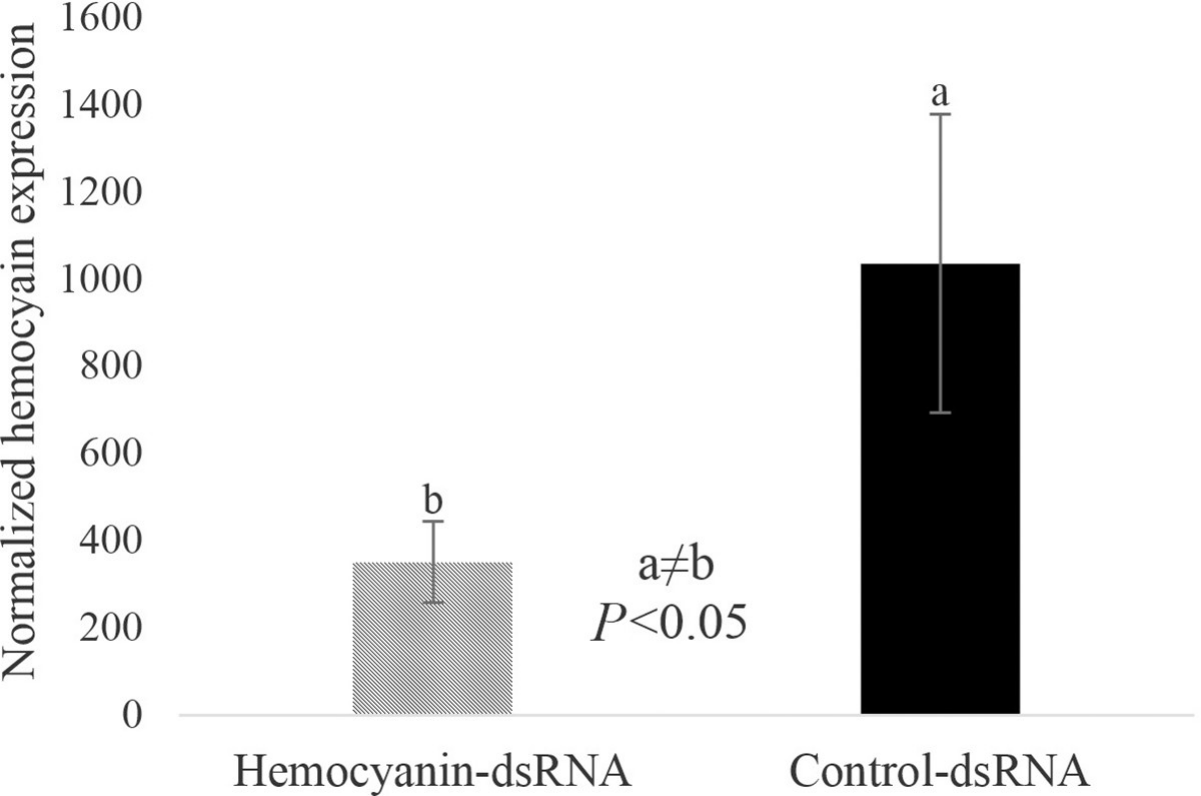
Hemocyanin-1 expression 10-days post injection in blue color morph *D*. *citri* adults. qPCR quantification of hemocyanin-1 (relative to α-tublin) in *D*. *citri* adults revealed significant reduction of hemocyanin-1 expression in dsRNA microinjected insects compared to control dsRNA injected insects.

To test whether changing expression of the *D*. *citri* hemocyanin-1 gene was sufficient to regulate *C*Las titer, we attempted RNA interference to silence hemocyanin-1. *D*. *citri* hemocyanin-1 was significantly reduced 10 days after microinjection of hemocyanin-1 dsRNA by 3-fold. The effect of dsRNA at concentrations of 50, 100 and 200 ng/μl was tested and identified that 100 ng/μl provided an optimal silencing effect for this gene, although a 3-fold reduction was the maximum level of transcript suppression achieved in all experiments. Microinjection of *D*. *citri* by dsRNA or water did not significantly affect the survival of the insects (not shown). The obtained Ct values were converted into normalized relative quantities (NRQs), and the yielded values of NRQs were used for statistical analysis. The expression of hemocyanin 10-days post microinjection was three-fold lower in insects injected with control dsRNA *(P* <0.05, **Fig 7**).

### 2-fold reduction of *C*Las titer in *D*. *citri* adults with a 3-fold reduction in hemocyanin-1 expression

**Fig 8:**
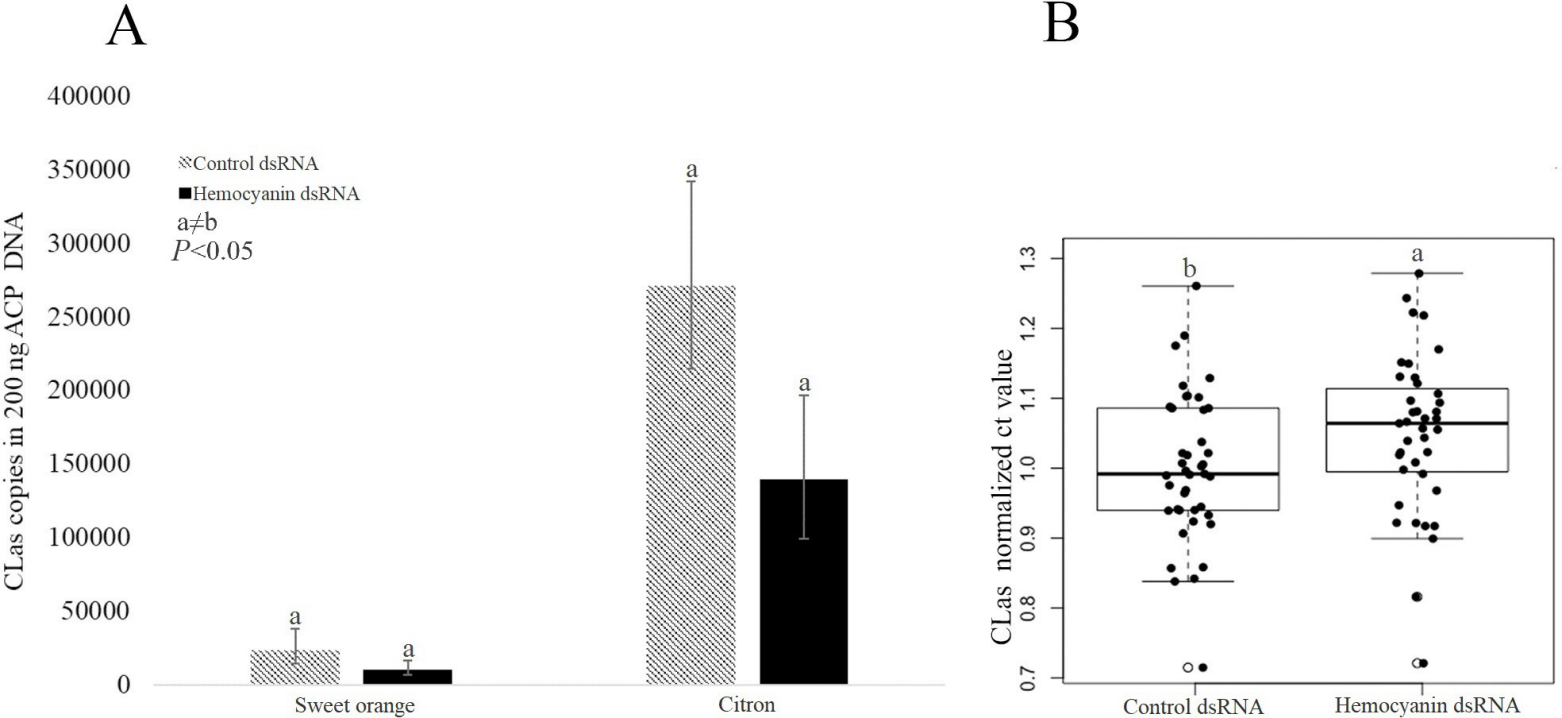
*C*Las titer 10-days post injection in blue color morph adult *D*. *citri*. (A) The copy number of *C*Las in hemocyanin silenced blue color morph reared on sweet orange and citron compared to non-silenced *D*. *citri*. Reduction in *C*Las titer in hemocyanin silenced insects was not statistically significant when data from different host plants are considered separately but was significant when normalized values of both host plants were considered together (B) Box plot analysis of normalized Ct values in control and hemocyanin dsRNA treated insects from both host plants. The increase in *C*Las qPCR Ct values in hemocyanin dsRNA treated insects represents a reduction in *C*Las titer. Sweet orange *D*. *citri*: n = 20; citron *D*. *citri*: n = 20.

The dsRNA microinjection experiments were carried out twice on both sweet orange-reared and citron-reared adult insects. In each of the four independent hemocyanin silencing experiments, *C*Las qPCR was used to estimate pathogen titer in the insect 10 days after injection of control dsRNA or hemocyanin dsRNA. In all four experiments, the average Ct value was slightly higher in hemocyanin silenced *D*. *citri*. As Ct value is inversely proportional to titer, these results indicate that hemocyanin silencing may be causing a reduction in *C*Las titer. However, due to large biological variation of Ct values among biological replicates, the difference in Ct values between treatments was not found to be statistically significant. Combined analysis of the effect of hemocyanin silencing on *C*Las titer across the four experiments required normalization because citron-reared *D*. *citri* had a higher *C*Las titer than sweet orange-reared *D*. *citri* (**Fig 8A**). For each of the four replicate experiments, the Ct values for each control dsRNA or hemocyanin dsRNA biological replicate were normalized by division by the average control Ct value for that experiment. The normalized Ct values for all four experiments were compared between control and hemocyanin silenced insects. The average normalized control silenced Ct value was 1, and the average normalized hemocyanin silenced Ct value was 1.05 +/- SEM, indicating that the *C*Las qPCR Ct value is 5% greater in hemocyanin silenced insects. This 5% increase in Ct value (from 26.9 to 28.1) can be attributed to a 2-fold decrease in *C*Las titer in the DNA isolated from hemocyanin silenced insects compared to the control (**Fig 8B**). Statistical comparison of the normalized Ct values for the four experiments between control and hemocyanin silenced insects using a two-tailed Student’s *t*-test indicated that the increase in *C*Las qPCR Ct value in hemocyanin-silenced insects is significant *(P*< 0.05).

## Discussion

Several studies have reported differences in life history traits between blue and non-blue *D*. *citri* color morphs [34–36]. In the context of vector biology, blue morphs have a greater propensity for long distance flight, and hence, a greater ability to spread *C*Las longer distances. Our study shows that the blue color *D*. *citri* morphs acquire less *C*Las as compared to non-blue *D*. *citri* color morphs. Accordingly, blue morphs transmit less *C*Las to plants as compared to non-blue color morphs. However, the tradeoff with enhanced flight capability may make the blue morphs a more important vector, economically, for the long distance spread of *C*Las among groves.

We found that non-blue color morphs also have significantly greater titers of the endosymbionts *Wolbachia* and *Profftella* as compared to blue insects from the same population. Past studies in aphids have shown that insect body color can be affected by many factors including temperature, host plant, light, symbionts, and natural enemies [37]. Additionally, endosymbionts and host plants [38,39] are known to affect the availability of nutrients to the insect harboring the endosymbionts. This variable nutrient availability has been shown, in turn, to affect insect fitness and polyphenism [37]. As *D*. *citri* exhibit polyphenism that is correlated with relative levels of endosymbiont titers, we hypothesize that endosymbionts such as *Wolbachia* and *Profftella* may mediate the production of nutrients and other defense related metabolites needed by the psyllid [38, 40], and that these differential levels of defense metabolites and nutrients may impact the psyllid’s immune response to infection by *C*Las. Alternatively, titers of all three bacteria may be coordinately regulated by the insect’s immune system.

*Wolbachia* was recently shown to infect the gut of *D*. *citri* [14], including co-infection of gut epithelial cells with *C*Las [41]. Additionally, *Wolbachia* encodes a small DNA-binding protein which suppresses holin promoter activity and the lytic cycle of SC1 prophage in *D*. *citri* [12], presenting the possibility that increased titers of *Wolbachia* may promote the multiplication of *C*Las by suppressing the transition of its prophage into the lytic phase. *Profftella* is a bacterial symbiont that resides in the syncytium of the bacteriome, a specialized organ which harbors the bacterial symbionts. *Profftella* produces an abundance of diaphorin, a polyketide of unknown function in the relationship with *D*. *citri* and with weak cytotoxic activity in animal cells [5]. Infection with *C*Las induces *Profftella* to increase the production of diaphorin and structurally similar polyketides [21]. Horizontal gene transfer of the LysE gene from *Profftella* to *C*Las supports the hypothesis that these two bacteria may have been co-localized in *D*. *citri* in such a way for horizontal gene transfer to occur.

Similar to the obligate bacterial symbionts, a number of gram-negative bacterial plant pathogens are also transmitted transovarially from mother to offspring in their insect vectors [42, 43]. Different species of *Liberibacter* are transmitted transovarially at high rates by other psyllid species, including ‘*Candidatus* Liberibacter psyllaurous’ [43], ‘*Candidatus* Liberibacter solanacearum’ [44] and ‘*Candidatus* Liberibacter americanus’ [45], but transovarial transmission of *C*Las has been reported at only very low levels in *D*. *citri*. *C*Las can be transmitted during mating from male to female but the reverse is not true [46], which may suggest that *C*Las accumulates at greater titer in the reproductive system of male compared to female insects. The immunocompetence handicap hypothesis predicts that insect steroid hormones mediate a trade-off between immunity and reproductive effort in male insects [47, 48]. Immune response is lower in males than females, typically against the same challenges [49]. Consistent with these findings, male *D*. *citri* sustain greater mitochondrial damage in the midgut tissue in insects reared on *C*Las-infected trees as compared to females [14, 41]. Alternatively, higher titers of the bacteria may be necessary for invasion of *C*Las into the ovaries.

Reliable detection methods are critical for the development of epidemiological models of the spread of HLB. Even though sensitive detection methods exist for *C*Las, the pathogen cannot be detected consistently in plants due to its uneven distribution and the latency period between inoculation and systemic infection. *C*Las detection in insects for monitoring and epidemiological studies can be a powerful alternative [50]. Challenges with monitoring *C*Las within a vector population include variation in bacterial acquisition, which our findings reveal are correlated with morphological features. The use of non-blue insects for detection may help to increase the likelihood of detecting *C*Las in field-collected psyllid samples, enabling a more accurate representation of the risk of disease spread. Our findings are in contrast to those of Hall [51] who did not observe significant difference in *C*Las titer between color morphs in a field study on sweet orange “Valencia” plants. This discrepancy may be due to variation between the responses of different *D*. *citri* genotypes to *C*Las, a consequence of adult age not being controlled and/or changes in the color of adults that occur over time. Our insects are classified for color morphology within one week after molting to adulthood, before developmental and reproductive changes in color morphology are likely to occur and while *C*Las titer is still relatively low in the insect (as compared to more mature adults). These hypotheses, derived from the research on growth-chamber *D*. *citri* colonies, must be tested using field-collected insects to validate the relevance of these findings for *C*Las detection.

Management programs heavily rely on conventional insecticides as the main component of HLB control. Excessive application of insecticides increases insecticide resistance and kills non-target and/or beneficial insects in citrus groves. Basic knowledge of vector transmission efficiency is critical for epidemiological modeling and establishing effective control methods which target the efficient transmitter. The color variation plays an important role in prey preference [52], pathogen transmission efficiency [53], selective mate choice by females [54], deterring predators [55], and choosing their habitat [56]. The color variations may have a phenotypical [57] or genetic background [56] and are affected by endosymbionts [7] or nutritional condition [58]. On one hand, most studies have been focused on the toxicity of insecticides on the overall population of *D*. *citri*. Blue colored insects are less efficient in acquisition and transmission and more resistant to pesticides. On the other hand, some insecticides suppress insect immunity [59], which may give *C*Las a greater opportunity for transmission by insects which survive insecticide treatment. These trade-offs may interfere with the effectiveness of control strategies that block transmission. Taken together, toxicity of insecticides for HLB management should be evaluated to avoid suppression of insect immunity.

Differences between insect color morphs have been correlated with variation in immune genes, including E4 and FE4 in the green peach aphid (*Myzus persicae*) [60], and CYP4 and hemocyanin in *D*. *citri* [14]. Hemocyanin has a role in immunity and defense in arthropods [23, 25]. Given the previous description of the role for hemocyanin in blue coloration of other invertebrates [61], the hypothesis that elevated levels of hemocyanin is correlated with blue abdominal color was tested. We determined that the blue abdominal coloration of *D*. *citri* is due to the presence of pigmented cells in the fat body of the insect. Compared to non-blue *D*. *citri*, results show that blue color morphs have increased levels of hemocyanin protein and elemental copper, which is consistent with our previous study showing increased hemocyanin gene expression in blue color morphs as compared to non-blue color morphs [20]. These results support the hypothesis that hemocyanin plays a defensive role, and may function to suppress *C*Las in the insect vector. *C*Las transmission may benefit from the existence of sub-groups within the vector population that do not mount an effective immune response against the pathogen, such as the non-blue color morphs. To further investigate the function of hemocyanin in *D*. *citri*, we microinjected adult insects with dsRNA sequences designed to silence hemocyanin expression, and evaluated the effect of silencing on *C*Las titer in the insect during acquisition. We hypothesized that reducing expression of this immunity gene would increase the susceptibility of *D*. *citri* to the pathogen. Surprisingly, *C*Las titers decreased slightly in response to hemocyanin silencing. One possible explanation for these contradictory results is that other *D*. *citri* immune components may be activated upon hemocyanin silencing through injection, leading to suppression of *C*Las acquisition and replication in the vector. Since hemocyanin is one of the most highly expressed proteins in *D*. *citri*, the three-fold reduction of hemocyanin transcript levels we obtained may not have been enough to observe a larger effect on titer. Hemocyanin protein levels after silencing was not examined. It is possible that silencing hemocyanin somehow caused an increase in hemocyanin protein levels or the protein turn-over rate of hemocyanin is slow relative to mRNA stability and production. Hemocyanin is expressed in nymphs and may play different roles in the nymph compared to the adult. The delivery of hemocyanin dsRNA to nymphs, where the immune response to *C*Las is notably distinct from adults [20, 41], may yield a different result. The possibility exists that the hemocyanin protein may have multiple, opposing functions in the regulation of *C*Las titer or that alternative splice variants have distinct functions in *C*Las titer regulation. Future research comparing proteome and/or transcriptome profiles of the different color morphs in response to *C*Las infection will help to test these hypotheses and to clarify the functional activities of hemocyanin-1 in *D*. *citri*.

## Supporting information

S1 Fig *C*Las titer in different color morphs of *D. citri* in citron-reared adult insects. *C*Las copy number per insect equivalent is ~3.6 fold higher in non-blue compared to blue *D*. *citri*, but this difference is not significant at *P =* 0.05.
(DOCX)

S1 Table The sequences of the double stranded RNA constructs used to silence *Diaphorina citri* hemocyanin and a control double stranded RNA targeting *Chinese sacbrood virus*, which should not silence any gene in *Diaphorina citri*. (DOCX)
