# Supplementary material for: Color morphology of Diaphorina citri influences interactions with its bacterial endosymbionts and ‘Candidatus Liberibacter asiaticus’
Source: PLoS One. 2019 May 16;14(5):e0216599. doi: 10.1371/journal.pone.0216599 (PMC6522040; doi:10.1371/journal.pone.0216599)
Supplement: S1 Fig — CLas copy number per insect equivalent is ~3.6 fold higher in non-blue compared to blue D. citri, but this difference is not significant at P = 0.05. (DOCX) [file pone.0216599.s001.docx]

**Figure S1**. CLas titer in different color morphs of *D. citri* in citron reared adults. CLas copy number per insect equivalent is ~3.6 fold higher in non-blue compared to blue *D. citri*, but this difference is not significant.
