# Supplementary material for: Color morphology of Diaphorina citri influences interactions with its bacterial endosymbionts and ‘Candidatus Liberibacter asiaticus’
Source: PLoS One. 2019 May 16;14(5):e0216599. doi: 10.1371/journal.pone.0216599 (PMC6522040; doi:10.1371/journal.pone.0216599)
Supplement: S1 Table — (DOCX) [file pone.0216599.s002.docx]

| **Table S1**. Sequence of dsRNA constructs used in this study | |
| --- | --- |
| Name of dsRNA | sequence |
| CSBV-negative control | GATTTGGCTATTTGGGTCCTGTCTCCATCATTTGGAATGTTTAAGGATATAAGGAAGTTTATAGCTACAGATGAGGACCTATCTAAACCAATTACTACGGAAGGGTCCTTATTATTGGCCCCAACTAATCGTAACCCAGTGCTTAAGGAACAGAGTATAGAGATACTGGGCCTACAAAATGAAATGCAAGTATCTGAGTTAAATGGCACAGTATTCTATGCGAGTGATGTAATTTGTTATGATTATTCACAACAGGGAGCTTGTGGATCTCTGTGCTTCTTGTCCCGCTCCCAAAGACCTATTGTGGGAATGCATTTTGCTGGTCGAGGTGAGGGGTCCTGTGGAGAAGGTTATGGGGTTATTTTAACTAAAGAGGCTATTGGGGATATTTTAGCATTGAAGTCTCAACCTGTGGTACAGTTGGAAGATTGGGAAGGACCCAGTTTAGAA |
| Dc_Hm1 | GGACGCAACTTTGCTTCCAACTTCAACCCTAACCGATTCCAGAACCCTCGAGAGGCTGAGCAATACATGAGAGCTTTCCAACAAGGTGAACTTCAGCAAAGAGGTGCTGCATTCTCTGTCCTGAACAACAACCAGCTGAACCAAGCTGTTCAACTCTTTGACCTGTTCTACTTCGCTAACGACTTCAACACTTTCTATCAAGCTGCTTGCTTTGCCAGAGACAACATCAATGAAGGCCAATTCGTGTATGCTTTCTACACTGCTATTGTTCAACGCCCCGACACCAAACACCTCTCTCTTCCTGCCATCTCTGAAGTTTACCCTCAACTCTTCGTCAAGGCAACAGTCATCAAACAAGCCCAAGATGCCGCTGCTCAAGGGCAACACAACTTCTACGCGAAGATGCATCACGCCGGACATCAAAGAGAGTGTCAAAACGAAAACAACATTGTGG |
| Dc_Hm2 | CCTATCTTCAACAAACTGTTGCCAGATACAACTTGGAACGTATGTCCAATGGTTTACCTCAGGTTCAACAAATCGAATACGATCAACCAATCCAACCTGGATTCAACCCTGAACTTCAATACGAAAACGGACAAGCCGTTCCCAACAGACCTGCAAACATTCGAATCAAAACCAAACAAGCCAATAACAACAACAATGAAAACAACAACGTTGGCAACTCTTTTGTCGAAACCATCAAGATGCATGAGCAGAGAATCATGGAGGCTATAGATTTGAATGCTATAAAAGACCCAATGGGATCTTCCAAGCCGATCAACCCCTTCTACGCCACCAACACTCTCACCAACTGTATTGAATCCAACGCTGACTGCCCCAACACCCAATATTATGGCTCCTTCTTTACTGAACTGCTCAAGCTTGTAGGAAGTGCTTCGGACG |
| Dc_Hm2R | CGGATGAACAGTGAAAAATACAACAAGAATAATAACAACAATAACGGACAGAGCAGCGCGATGGAGTACTACTTCTACGGAAATAATATGAATAACATGAACATGAACAATATGAATAACAACAACAATTGGAATAACATGAACATGAATAATTGGAATAACATGAACAACAATTGGAACAATATGAACCGAAATAACATGAACAACATGAATAATTGGAATAACATGAACAACAATTGGAATAACATGAACAACAATTGGAATAACATGAACCGAAATAATTGGAACAACATGAACCGAAATAACATGAACAACATGAATAATTGGAATAACATGAACAACAATTGGAACAATATGAATAACAACTACAACAATGGC |
